# Identification of CTL Epitopes on Efflux Pumps of the ATP-Binding Cassette and the Major Facilitator Superfamily of *Mycobacterium tuberculosis*

**DOI:** 10.1155/2021/8899674

**Published:** 2021-01-05

**Authors:** Yan Lin, Yu Dong, Yanfeng Gao, Ranran Shi, Yubing Li, Xiuman Zhou, Wenwen Liu, Guodong Li, Yuanming Qi, Yahong Wu

**Affiliations:** School of Life Sciences, Zhengzhou University, Zhengzhou 450001, China

## Abstract

Tuberculosis is the world's most deadly infectious disease, with 10 million people falling ill and 1.5 million people dying from the disease every year. With the increasing number of drug-resistant *Mycobacterium tuberculosis* (MTB) strains and prevalence of coinfection of MTB with human immunodeficiency virus, many challenges remain in the prevention and treatment of tuberculosis. Therefore, the development of safe and effective tuberculosis vaccines is an urgent issue. In this study, we identified cytotoxic T lymphocyte epitopes on drug resistance-associated membrane protein efflux pumps of MTB, the ATP-binding cassette and the major facilitator superfamilies. First, three online software were used to predict HLA-A2-restricted epitopes. Then, the candidate epitopes were confirmed with the T2A2 cell binding affinity and peptide/MHC (pMHC) complex stability assays and *in vitro* immune activity experiments. Two drug-resistant T lymphocyte epitopes, designated Rv1218c-p24 and Rv2477c-p182, were selected, and their immunogenic activities studied *in vivo* in genetically engineered mice. The immune activities of these two epitopes were improved with the help of complete Freund's adjuvant (CFA). The epitopes identified here provide a foundation for the diagnosis and treatment of patients infected with drug resistant and the future development of a multiepitope vaccine.

## 1. Introduction

Despite the extensive use of the *Mycobacterium bovis* (Mb) bacilli Calmette Guérin (BCG) vaccine, tuberculosis (TB) is still the world's most deadly infectious disease. The World Health Organization (WHO) estimated that 10 million cases occurred worldwide in 2018, and 1.5 million people died from this disease, including 251,000 people coinfected with human immunodeficiency virus. Two-thirds of the total number of new cases of TB occurred in eight countries, including India, China, and Indonesia [1, 2]. Although antibiotics such as rifampicin, isoniazid, and ofloxacin are recommended by the WHO for the treatment of tuberculosis, and these antibiotics have significantly prevented the spread of MTB in past decades, they are associated with toxicities and may hasten the development of drug resistance in MTB, so the number of new cases remains huge [3]. Drug-resistant TB (DR-TB) can be divided into multidrug-resistant TB (MDR-TB), extensively drug-resistant TB (XDR-TB), and totally drug-resistant TB (TDR-TB). MDR-TB causes a disease that does not respond to the two most powerful first-line anti-TB drugs, isoniazid and rifampicin, where XDR-TB is a more serious form of MDR-TB, which does not respond to the most effective second-line anti-TB drugs and often leaves patients with no further appropriate medicines [3–5]. BCG is the only vaccine currently available that effectively prevents MTB, but BCG only induces a good protective immune response in childhood [6]. Therefore, the development of new strategies for the treatment and/or prevention of MDR-TB and XDR-TB is urgently required.

In recent years, immunotherapy has developed rapidly and demonstrated its unique advantages in the treatment of many diseases, including cancer and infectious diseases, and the CD8^+^ T cell-mediated cellular immune response plays an important role in these therapies. Several studies have reported that reactive CD8^+^ T cells can be detected in the circulation of purified protein derivative-positive (PPD^+^) healthy donors and patients with active TB, which are responsive to the bacterial proteins ESAT-6, CFP-10, CFP21, and so on [7–10]. Several groups of MHC I-restricted cytotoxic T lymphocyte (CTL) epitopes have been identified in MTB, and a key research strategy is to select ideal antigens of MTB to identify the CTL epitopes.

The main reason for the emergence of DR-TB is that MTB uses many mechanisms to eliminate antibiotics, including drug efflux pumps and simulating targets [11]. Antibiotic efflux is a ubiquitous mechanism underlying both innate and acquired drug resistance. So far, five efflux pump families have been identified: the ATP-binding cassette (ABC) superfamily [12], the major facilitator superfamily (MFS) [13], the multidrug and toxic compound extrusion family (MATE) [14], small multidrug-resistant family (SMR) [15], and the resistance nodulation division family (RND) [16]. ABC transporters and MFS are the largest superfamilies of efflux pumps in *M. tuberculosis*. The overexpression of some ABC transporter proteins is reported to be an important cause of drug resistance, ultimately leading to chemotherapy failure. Rv0194, Rv0933 (PstB), Rv1218c, Rv1819c, Rv2209, Rv2477c, Rv2686c, Rv2687c, Rv1273C, and Rv2937 have been identified as members of the ABC transporter superfamily that are associated with MDR-TB and XDR-TB [17–19]. The increased expression of Rv0933 (PstB) in clinical drug-resistant tuberculosis isolates may contribute to drug resistance in MTB [20], and the significantly elevated expression of Rv1218c at the transcriptional level has been observed in MDR-TB strains [21]. Overexpression of two of the proteins (Rv2477 and Rv2209) has also been observed with ofloxacin stress in MTB [22]. The deletion of the Rv1877 homolog increased bacterial susceptibility to ethidium bromide, acriflavine, and erythromycin [23]. The MFS transporter family is the largest class of proteins present in all living organisms. In general, MFS transporters are efflux pumps that transport small molecular solutes along electrochemical gradients [24]. Some MFS transporters can also form triple complexes with a membrane fusion protein and an outer membrane protein, across the outer membrane of Gram-negative bacteria. Rv2994, Rv3728, Rv2459c, Rv1877, Rv1258c, and Rv1410c are drug resistance-related efflux pumps in the MFS' family expressed in MTB [10, 23, 25, 26].

In this study, HLA-A^∗^0201-restricted CD8^+^ CTL epitopes were predicted on 10 efflux pump antigens with the online tools SYFPEITHI (http://www.syfpeithi.de/), BIMAS (https://www-bimas.cit.nih.gov/molbio/hla_bind/hla_motif_search_info.html), and NetCTL-1.2 (http://www.cbs.dtu.dk/services/NetCTL/), and the epitopes with high scores were selected and synthesized. Peptide binding affinity and peptide/HLA-A^∗^0201complex stability assays were performed using T2A2 cells *in vitro*, and the abilities of peptide induced CTLs (*in vitro* and *in vivo*) to secrete interferon *γ* (IFN-*γ*) and lyse the target cells that were detected.

## 2. Materials and Methods

### 2.1. Candidate Peptides

First, we used the common online T cell epitope prediction tools, SYFPEITHI, BIMAS, and NetCTL-1.2, to predict candidate 9-mer peptides from 10 antigens of ABC/MFS Efflux Pumps Family [27, 28]. Then, these peptides were synthesized by standard solid-phase Fmoc strategy and purified with reverse-phase high performance liquid chromatography (RP-HPLC) (the purity of each peptide is more than 95%). Subsequently, the molecular weight of each peptide was confirmed with electrospray ionization mass spectrometry (ESI-MS). Peptide cyclooxygenase-2 (COX-2)_321-329_ (ILIGETIKI) which identified before was used as positive control in T2A2 binding assay and peptide/HLA-A^∗^0201 complex stability assay [29]. The IA^b^-restricted HBV core antigen-derived T helper epitope (sequence 128-140: TPPAYRPPNAPIL) was used in the *in vivo* assay [30]. All the purified peptide with the correct molecular weight were dissolved at the concentration of 10 mg/mL in DMSO and stored at -80°C.

### 2.2. Cell Line, Human Blood Samples, and Transgenic Mice

The lymphoblastic cell line T2A2 cells (TAP deficient) were a kindly gift by Professor Yuzhang Wu (Third Military Medical University, China) and were cultured in IMDM medium supplemented with 20% fetal bovine serum (FBS, Biological Industries, BI, Israel), 2 mM L-glutamine, 100 units/mL penicillin, and 100 *μ*g/mL streptomycin, at 37°C in a humidified atmosphere of 5% CO_2_.

40 mL peripheral blood samples were obtained from HLA-A^∗^02-positive PPD-positive (HLA-A^∗^02^+^PPD^+^) and HLA-A^∗^02-positive PPD-negative (HLA-A^∗^02^+^PPD^−^) donors, and verbal informed consent was obtained from all individuals, and the collection was approved by the ethics committee of Zhengzhou University; then, PBLs (peripheral blood lymphocytes) were isolated by using Ficoll-Paque density gradient centrifugation method (lymphocyte separation liquid, TBD, China).

HLA-A2.1/K^b^ transgenic mice used in this article were purchased from Model Animal Research Center of Nanjing University (Nanjing, China) and bred in our lab in specific pathogen-free facilities (SPF) with SPF food and sterilized water [31]. And mice used in *in vivo* experiment were 8 weeks; all experiments were performed according to the guidelines of the Institutional Animal Care and Use Committee of Zhengzhou University.

### 2.3. Binding Affinity and Peptide/HLA-A2 Complex Stability Assay

Candidate peptides have high binding affinity to MHC-I molecule and form a stable peptide/MHC-I complex which is the first step to activate the specific T cell response. So, these assays were performed according to our former study [32]. Briefly, T2A2 cells (1 × 10^6^ cells/mL) resuspended in serum-free IMDM medium were seeded in 24-well plates, added 50 *μ*g/mL peptide and 3 *μ*g/mL human *β*2-microglobulin (*β*2-M, Merck, Germany) simultaneously, and cultured at 37°C for 18 h. Then, cells were collected, washed twice with precooled PBS buffer (containing 5% FBS), and incubated with HLA-A2-PE-Cyanine 7 monoclonal antibody (Clone: BB7.2, 25-9876-42, eBioscience, USA) for 30 min at 4°C. Finally, cells were harvested, washed, and analyzed the fluorescence intensity (MFI) of each group by flow cytometer (FACS Calibur, BD Bioscience, USA). The fluorescence index (FI) was calculated as follows: FI = [mean fluorescence intensity (MFI) of the peptide − MFI of the background]/[MFI of the background]. And the background group was added with the corresponding solution buffer.

The peptide/MHC complex stabilization assays were performed similarly [32]. T2A2 cells were incubated with 50 *μ*g/mL peptides and 3 *μ*g/mL *β*2-M for 18 h. Then, 10 *μ*g/mL brefeldin A (203729, Sigma, USA) were added in medium for another 1 h, subsequently washed by serum-free IMDM medium and incubated at 37°C for 0, 2, 4, and 6 h. Finally, cells were washed twice, stained with HLA-A2-PE-Cyanine 7 monoclonal antibody, and analyzed by flow cytometer (FACS Calibur, BD Bioscience, USA). DC_50_ was defined as an estimate of the time required for the loss of 50% of the peptide/HLA-A^∗^0201 complexes stabilized at time 0 h.

### 2.4. Generation of Autologous DCs from Human PBMCs

Briefly, PBMCs isolated from HLA-A2^+^ PPD^+^ and HLA-A2^+^ PPD^−^ donors were resuspended in RPMI-1640 medium supplemented with 10% FBS and seeded in 6-well plates for 4 h at 37°C, 5% CO_2_. Adherent cells were washed twice with RPMI-1640 medium, and then cultured in DC medium (RPMI-1640 medium supplement with 10% FBS, 2 mM L-glutamine, 100 units/mL penicillin, and 100 *μ*g/mL streptomycin, 100 ng/mL IL-4 (200-04, PeproTech), 100 ng/mL GM-CSF (300-03, PeproTech)). On day 3, Fresh DC media (100 ng/mL IL-4 and 100 ng/mL GM-CSF) were replenished. On day 5 of the culture, 10 ng/mL lipopolysaccharide (LPS, Sigma, USA) was added for another two days to induce the maturation of DCs [33].

### 2.5. Induction of Peptide Specific CTLs *in vitro*

The CTL induction assay was performed in accordance with the protocols as previously described [32, 33]. Briefly, DCs were pulsed with different peptides at a final concentration of 10 *μ*g/mL in the presence of 3 *μ*g/mL *β*2-M for 24 h. Then, peptide-pulsed DCs (1 × 10^5^) were cocultured with thawed autologous T cells (1 × 10^6^) in 24-well plates in RPMI-1640 medium supplement with 10% FBS, human recombinant IL-2 (rhIL-2, 50 U/mL, 200-02, PeproTech, USA), and human recombinant IL-7 (rhIL-7, 10 ng/mL, 200-07, PeproTech, USA). Autologous T cells were restimulated three times at weekly intervals to generate peptide-specific CTLs. 50 U/mL rhIL-2 and 10 ng/mL rhIL-7 were added at intervals of 2 days. On day 21, the induced T cells were collected and their IFN-*γ* secretion was determined with ELISPOT assay, intracellular cytokine staining (ICS) assay, and LDH cytotoxic assay.

### 2.6. Generation of CTLs in HLA-A2.1/K^b^ Transgenic Mice

Activity of CTLs induced with IFA-emulsified peptide in HLA-A2.1/K^b^ transgenic mice was performed as previously described [31]. Firstly, HLA-A2.1/K^b^ transgenic mice (*n* = 5) were subcutaneously immunized at the base of tail with each peptide (100 *μ*g/mice) and T helper epitope (140 *μ*g/mice) emulsified with incomplete Freund's adjuvant (IFA, F5506, Sigma, USA) at 1 : 1 three times (on days 0, 5, and 10).

To establish a similar immune effect of PPD^+^ donor, we considered whether the complete Freund's adjuvant (CFA, F5881, Sigma, USA) which contains inactivated mycobacterium tuberculosis could use for enhancing the immune response of the peptide. Activity of CTLs induced with peptide after preimmunization with CFA in HLA-A2.1/K^b^ transgenic mice was performed. Emulsified complete Freund's adjuvant (CFA, 200 *μ*L) was subcutaneously injected into HLA-A2.1/K^b^ transgenic mice (*n* = 5) on day-14. The mice were then subcutaneously immunized at the base of the tail with each peptide (100 *μ*g/mice) and T helper epitope (140 *μ*g/mice) emulsified with incomplete Freund's adjuvant (IFA) at 1 : 1 (on days 0, 5, and 10).

On day 11, all mice were sacrificed and the serum was collected from each mouse to measure the IFN-*γ* concentration with an ELISA assay. The splenic lymphocytes were isolated from each mouse and restimulated once with each peptide (10 *μ*g/mL) and murine recombinant IL-2 (rmIL-2, 50 U/mL, 212-12-20, PeproTech, USA) *in vitro* for another 6 days. On day 7, the IFN-*γ* release in each group was measured with an intracellular cytokine staining (ICS) assay and the lysis activity was measured with an LDH assay, and the E : T ratios were 20 : 1, 40 : 1, and 80 : 1. The body weight change of each mouse was recorded [31].

### 2.7. Enzyme-Linked Immunospot (ELISPOT) Assay

ELISPOT assay was performed using a commercial kit according to previous studies (Dakewe, China) [32]. In brief, on day 21, peptide-induced T cells were used as effector cells, and effector cells (1 × 10^5^) and stimulator target cells (T2A2 cells loaded with/without corresponding peptide, 1 × 10^5^) were seeded into the 96-well plates which were precoated with anti-human IFN-*γ* antibody and coincubated at 37°C. After incubation for 18 h, cells in plate were lysed by ice deionized water and washed thrice by provided buffer of the kit, followed by incubating with detection antibodies and enzyme-linked avidin every other hour at 37°C. After the termination of drying, the number of spots was determined automatically by using a computer-assisted video image analyzer (Dakewe, China).

### 2.8. Intracellular Cytokine IFN-*γ* Staining (ICS) Assay

IFN-*γ* release of induced CD8^+^ T cells was measured by the intracellular cytokine staining assay according to our former study [31, 34]. Briefly, the induced CTLs (1 × 10^6^) and T2A2 cells loaded with/without corresponding peptide (1 × 10^6^) were incubated by adding protein transport inhibitor (containing brefeldin A) for 5 hours at 37°C. Afterward, cells were harvested, transferred to 1.5 mL EP tubes, and washed by PBS (contain 2% FBS). Cells were then stained for cell surface markers (anti-human CD3 PerCP-eFlour (Clone: OKT3, 46-0037-42, eBioscience, USA)/anti-mouse CD3 PerCP-eFlour710 (Clone: 17A2, 46-0032-80, eBioscience, USA) and anti-human CD8*α* APC (Clone: SK1, 17-0087-42, eBioscience, USA)/anti-mouse CD8*α* PE (Clone: 53-6.7, 12-0081-82, eBioscience, USA)) for 30 min at 4°C. Afterward, the cells were fixed by fixation buffer for another 30 min at room temperature and washed twice with permeabilization wash buffer. Anti-human IFN-*γ* PE (Clone: 4S.B3, 12-7319-42, eBioscience, USA) or anti-mouse IFN-*γ* APC (Clone: XMG1.2, 17-7311-81, eBioscience, USA) was added to those tubes for intracellular staining for 30 min at 4°C. After being washed twice with permeabilization wash buffer, the IFN-*γ* release of CD8^+^ T cells was analyzed by flow cytometry (FACS Calibur, BD Bioscience, USA).

### 2.9. ELISA Assay

ELISA assay was performed using a commerciall kit following the manufacturer's instructions (121002, Dakewe, China) [34, 35]. In brief, the serum of each mouse was diluted and added into the 96-well plates which were precoated with anti-human IFN-*γ* antibody and coincubated at room temperature, then incubated with detection antibodies at room temperature for 90 min, washed quartic, and added enzyme-linked avidin every other hour. After adding TMB and stop solution, the OD450 was detected and the concentration of IFN-*γ* in each group was calculated.

### 2.10. LDH Cytotoxicity Assay

Cytotoxic activity was tested based on the measurement of LDH release using the nonradioactive cytotoxicity assay kit (G1780, Promega, USA) at gradient E : T (effector cells/target cells) ratio [32]. T2A2 cells (2 × 10^6^/mL) were pulsed by each peptide (50 *μ*g/mL) at 37°C for 4 h and used as the target cells. CTLs induced by each group were used as the effector cells. Then, target cells (1 × 10^5^/well) were cocultured with different amount effector cells (E : T ratio was 12.5 : 1, 25 : 1, and 50 : 1 *in vitro* and 20 : 1, 40 : 1, and 80 : 1 *in vivo*, respectively) at 37°C for 4 h. Subsequently, the plate was centrifuged, and the supernatant of each well was transferred to a new plate, added the substrate mixture, and incubated out of light at room temperature. Finally, the stop solution was added and OD490 was measured. The percentage of specific lysis of the target cells was determined as follows: percentage of specific lysis = [(experimental release − effector spontaneous release − target spontaneous release)/(target maximum release − target spontaneous release)] × 100.

### 2.11. Statistical Analysis

All data were presented as means ± SD. Comparisons between experimental groups and relevant controls were analyzed by 1-tailed Student's *t*-test. *P* < 0.05, *P* < 0.01, and *P* < 0.001 represent significance of differences relative to the control group.

## 3. Results

### 3.1. CTL Epitope Prediction and Synthesis

First, 29 HLA-A^∗^0201-resticted candidate 9-mer peptides with high prediction scores (SYFPEITHI > 24, BIMAS > 20, and NetCTL − 1.2 > 0.9) were synthesized, as shown in Table 1, and purified with RP-HPLC, and their molecular weights were confirmed with ESI-MS (Table 2).

### 3.2. Binding Affinity and Peptide/HLA-A^∗^0201 Complex Stability

**Figure 1:**
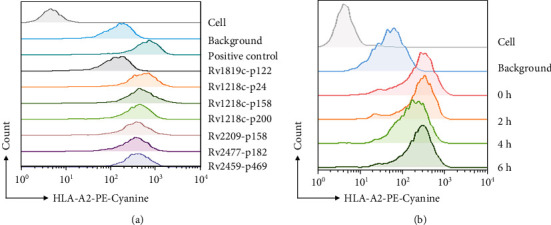
Representative flow chart of binding affinity and peptide/HLA-A2 complex stability assay. Representative flow chart of (a) the binding affinity of the peptides and (b) peptide/HLA-A2 complex stability assay of peptide Rv2477c-p182.

Because the solubility of some peptides was low, the binding affinity of only 17 peptides was determined, as shown in Table 2 and Figure 1(a), and 14 peptides with high binding affinity (FI > 1.5) were selected to measure the stability of their peptide/HLA-A^∗^0201 complexes. Six peptides, Rv1218c-p24, Rv1218c-p200, Rv1218c-p158_,_ Rv2209-p158, Rv2477c-p182, and Rv2459c-p469, formed stable peptide/HLA-A^∗^0201 complexes (DC_50_ > 4 h) (Figure 1(b)) and were selected for further study.

### 3.3. IFN-*γ* Secretion of Peptide-Induced CTLs *in vitro*

**Figure 2:**
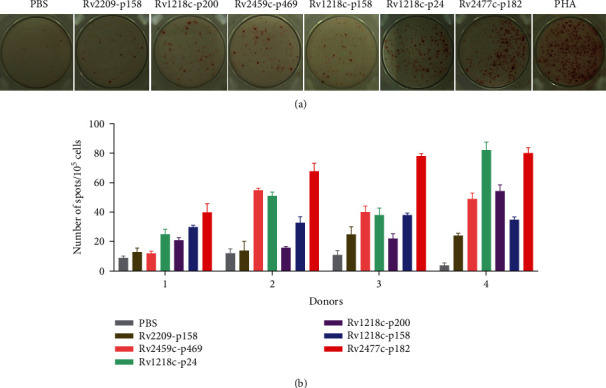
IFN-*γ* secretion of peptide-induced CTLs was determined with ELISPOT assay. Candidate peptides were used to induce CTLs with peptide-loaded DCs from four healthy PPD^+^ donors *in vitro*. T cells were stimulated three times at weekly intervals, and rhIL-2 (50 U/mL) and rhIL-7 (10 ng/mL) were added at intervals of 2 days. On day 21, the induced T cells were collected to measure IFN-*γ* secretion with an ELISPOT assay. (a) Typical chart for each group. (b) Statistical analysis of each peptide in four donors (*n* = 4). Peptide-loaded T2A2 cells or T2A2 cells only were used as the stimulated cells. PBS group was the negative control; PHA was used as the positive control.

**Figure 3:**
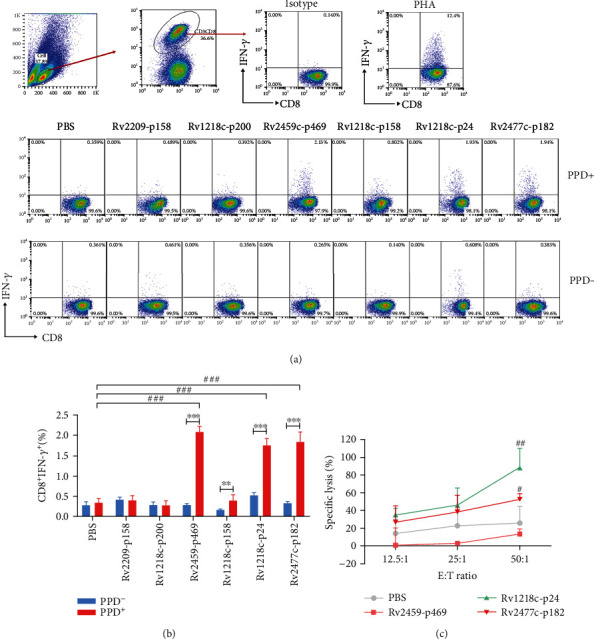
Immune activity of peptide-induced CTLs *in vitro* was determined with intracellular cytokine staining (ICS) assay and LDH assay. CTLs were induced *in vitro* with peptide-loaded DCs from three healthy PPD^+^ donors and three PPD^−^ donors. T cells were stimulated three times at weekly intervals, and rhIL-2 (50 U/mL) and rhIL-7 (10 ng/mL) were added at intervals of 2 days. On day 21, the induced T cells were collected and their IFN-*γ* secretion and lysis activity were measured with intracellular cytokine staining (ICS) and LDH assays. (a) Typical flow chart for each group of PPD^+^ and PPD^−^ donors. (b) Statistical results of ICS assay for each peptide (*n* = 3). (c) LDH assay (*n* = 4). Peptide-loaded T2A2 cells were used as the stimulated cells and target cells. PBS group was the negative control; PHA was used as the positive control. Statistical significance was determined with Student's *t*-test. ^∗^*P* < 0.05, ^∗∗^*P* < 0.01, and ^∗∗∗^*P* < 0.001 represent the significance of the differences between the PPD^+^ donors and PPD^−^ donors. ^#^*P* < 0.05, ^##^*P* < 0.01, and ^###^*P* < 0.001 represent the significance of the differences between the peptide-treated groups and the PBS group.

First, IFN-*γ* secretion of peptide-induced CTLs from four HLA-A^∗^02^+^PPD^+^ donors was measured with an ELISPOT assay (Figures 2(a) and 2(b)). The results showed that the Rv1218c-p158-, Rv2459-p469-, Rv1218c-p24-, and Rv2477c-p182-induced CTLs secreted IFN-*γ* most potently in at least three of the five PPD^+^ donors. To determine whether these peptides could distinguish between HLA-A^∗^02^+^PPD^+^ donors and HLA-A^∗^02^+^PPD^−^ donors, we performed an intracellular cytokine staining (ICS) assay. As shown in Figures 3(a) and 3(b), the Rv2459c-p469-, Rv1218c-p24-, Rv1218c-p158-, and Rv2477c-p182-induced CTLs from HLA-A^∗^02^+^PPD^+^ donors contained significantly more CD8^+^ IFN-*γ*^+^ T cells than those from HLA-A^∗^02^+^PPD^−^ donors. Furthermore, the Rv2459c-p469-, Rv1218c-p24-, and Rv2477c-p182-induced CTLs from HLA-A^∗^02^+^PPD^+^ donors contained more CD8^+^ IFN-*γ*^+^ T cells than the PBS-treated group. Therefore, Rv2459c-p469, Rv1218c-p24, and Rv2477c-p182 were selected to assess their cytotoxic activity *in vitro* in HLA-A^∗^02^+^PPD^+^ donors.

### 3.4. *In vitro* Cytotoxic Activity of Peptide-Induced CTLs

An LDH cytotoxicity assay was used to determine whether the CTLs induced by these three selected peptides lysed the target cells. PBMCs from HLA-A^∗^02^+^PPD^+^ donors were stimulated with each candidate peptide as described above. T2A2 cells pulsed with the corresponding peptide were used as the target cells. As shown in Figure 3(c), the Rv1218c-p24 and Rv2477c-p182 peptide-induced CTLs lysed the target cells at an E : T ratio of 50 : 1, and the lysis rates were 88.6% and 52.5%, respectively, which differed significantly from that of the PBS group. Therefore, these two peptides (Rv1218c-p24 and Rv2477c-p182) were selected for an *in vivo* assay.

### 3.5. Activity of CTLs Induced with IFA-Emulsified Peptide in HLA-A2.1/K^b^ Transgenic Mice

**Figure 4:**
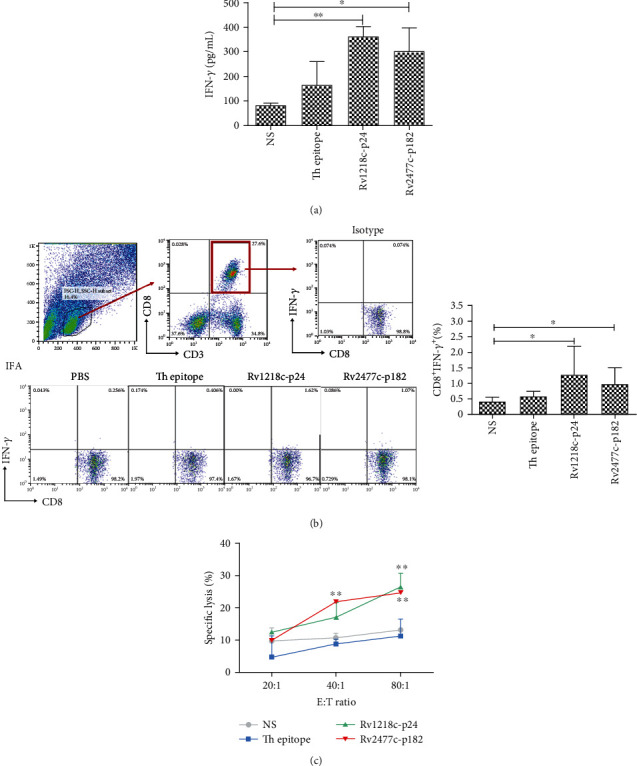
Activity of CTLs induced with IFA-emulsified peptide in HLA-A2.1/K^b^ transgenic mice. HLA-A2.1/K^b^ transgenic mice (*n* = 5) were subcutaneously immunized at the base of the tail with each peptide (100 *μ*g/mice) and T helper epitope (140 *μ*g/mice) emulsified with incomplete Freund's adjuvant (IFA) at 1 : 1, three times (on days 0, 5, and 10). On day 11, all the mice were sacrificed and the serum was collected from each mouse to measure the IFN-*γ* concentration with an ELISA assay (a). The splenic lymphocytes were isolated from each mouse and restimulated once with each peptide (10 *μ*g/mL) and mIL-2 (10 U/mL) *in vitro* for another 6 days. On day 7, the IFN-*γ* release in each group was measured with an intracellular cytokine staining (ICS) assay (b) and the lysis activity was measured with an LDH assay (c). Peptide-loaded T2A2 cells were used as the target cells. Normal saline (NS) group and Th epitope only group were the negative controls. Statistical significance was determined with Student's *t*-test (*n* = 5). ^∗^*P* < 0.05 and ^∗∗^*P* < 0.01 represent the significance of differences relative to the NS group.

To investigate whether the selected peptides induced CTLs in HLA-A2.1/K^b^ mice, HLA-A2.1/K^b^ transgenic mice (*n* = 5) were immunized subcutaneously at the base of the tail with each peptide (100 *μ*g/mice) and T helper epitope (140 *μ*g/mice), emulsified with incomplete Freund's adjuvant (IFA) at a ratio of 1 : 1, three times (on days 0, 5, and 10). On day 11, the serum was collected from each mouse to determine the IFN-*γ* concentration with an ELISA, and the results are shown in Figure 4(a). The Rv1218c-p24- and Rv2477c-p182-induced T cells produced significantly more IFN-*γ* (363 pg/mL and 303.5 pg/mL, respectively) than the NS group. The splenic lymphocytes were isolated from each mouse and stimulated once with each peptide (10 *μ*g/mL) and mIL-2 (50 U/mL) *in vitro* for 6 days. On day 7, the IFN-*γ* release in each group was measured with an intracellular cytokine staining (ICS) assay (Figure 4(b)). We found that the Rv1218c-p24 and Rv2477c-p182 groups induced more CD8^+^IFN-*γ*^+^ T cells than the NS group. In a lysis assay (Figure 4(c)), the lysis rate of the Rv1218c-p24 group at an E : T of 80 : 1 was 26.4%, which differed significantly from that of the NS group, and the lysis rates of the Rv2477c-p182 group at E : T of 40 : 1 and 80 : 1 were 21.8% and 24.6%, respectively, which also differed significantly from that of the NS group.

### 3.6. Activity of CTLs Induced with Peptide after Preimmunization with CFA in HLA-A2.1/K^b^ Transgenic Mice

**Figure 5:**
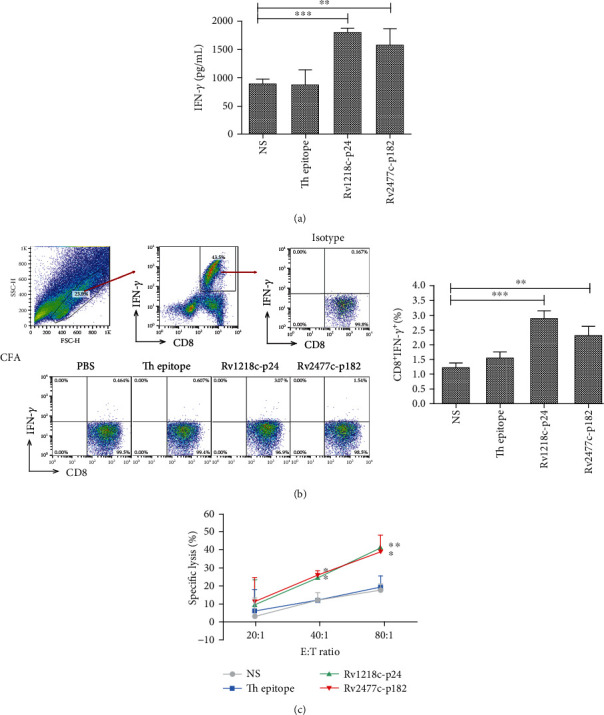
Activity of CTLs induced with peptide after preimmunization with CFA in HLA-A2.1/K^b^ transgenic mice. Complete Freund's adjuvant (CFA, 200 *μ*L) was injected subcutaneously into HLA-A2.1/K^b^ transgenic mice (*n* = 5) on day-14. The mice were then subcutaneously immunized at the base of the tail with each peptide (100 *μ*g/mice) and T helper epitope (140 *μ*g/mice) emulsified with incomplete Freund's adjuvant (IFA) at 1 : 1 three times (on days 0, 5, and 10). On day 11, all mice were sacrificed and the serum was collected from each mouse to measure the IFN/-*γ* concentration (a). The splenic lymphocytes were isolated from each mouse and restimulated once with each peptide (10 *μ*g/mL) and mIL-2 (10 U/mL) in vitro for another 6 days. On day 7, the IFN-*γ* release in each group was measured with an intracellular cytokine staining (ICS) assay (b) and the lysis activity were measured with an LDH assay (c). Peptide-loaded T2A2 cells were used as the target cells. Normal saline (NS) group and Th epitope only group were the negative controls. Statistical significance was determined with Student's *t*-test (*n* = 5). ^∗^*P* < 0.05, ^∗∗^*P* < 0.01, and ^∗∗∗^*P* < 0.001 represent the significance of differences relative to the NS group.

The results of our *in vitro* and *in vivo* experiments indicated that these two peptides, Rv1218c-p24 and Rv2477c-p182, induced the strongest immune response in PPD^+^ donors. Therefore, we investigated whether MTB in the complete Freund's adjuvant (CFA) could be used to mimic the symptoms of PPD^+^ donors to enhance the immune response to the peptide. Emulsified complete Freund's adjuvant (CFA, 200 *μ*L) was subcutaneously injected into HLA-A2.1/K^b^ transgenic mice (*n* = 5) on day-14. The mice were then immunized subcutaneously at the base of tail with each peptide (100 *μ*g/mice) and T helper epitope (140 *μ*g/mice) emulsified with incomplete Freund's adjuvant (IFA) (1 : 1) three times (on days 0, 5, and 10). On day 11, the serum was collected from each mouse to detect the IFN-*γ* concentration with an ELISA assay, and the results are shown in Figure 5(a). The peptides Rv1218c-p24 and Rv2477c-p182 triggered the production of significantly more IFN-*γ* (1817.0 pg/mL and 1587.8 pg/mL, respectively) than was produced in the NS group. The splenic lymphocytes isolated from each mouse were then stimulated once with each peptide (10 *μ*g/mL) and mIL-2 (50 U/mL) in vitro for 6 days. On day 7, the IFN-*γ* release in each group was measured by intracellular cytokine staining (ICS) assay (Figure 5(b)). We found that the Rv1218c-p24 and Rv2477c-p182 groups produced more CD8^+^ IFN-*γ*^+^ T cells than the NS group. In the lysis assay (Figure 5(c)), the lysis rates of the Rv1218c-p24 group at E : T of 40 : 1 and 80 : 1 were 24.6% and 41.2%, respectively, which were significantly higher than that of the NS group, and the lysis rates of Rv2477c-p182 group at E : T of 40 : 1 and 80 : 1 were 25.8% and 38.9%, respectively, which were also significantly higher than that of the NS group. The combined results shown in Figures 4 and 5 indicate that preimmunization with CFA before peptide immunization enhanced the immune response of the peptide.

## 4. Discussion

Tuberculosis (TB) is an infectious disease caused by MTB that remains a major cause of death worldwide. With the development of genomic techniques, increasing numbers of proteins associated with the drug resistance of MTB have been identified. However, the prevalence of MDR-TB, XDR-TB, and TDR-TB infections is increasing alarmingly in many countries. Only 56% of patients with MDR-TB can be treated successfully, and there are no appropriate medicines for treating XDR-TB [36]. To meet the evolving challenge of TB, new anti-TB drugs and vaccines against drug-resistant TB are urgently required.

Studies have shown that the efflux systems play an important role in MTB drug resistance, and five efflux pump families and many efflux pump proteins have been shown to be associated with it. In recent years, immunotherapy has played an important role in fighting tumors and infectious diseases. CD8^+^ T lymphocytes, which are activated by the epitopes of target antigens, are among the most important immune cells and play a critical role in cell-mediated immunity to control latent MTB infections. CD8^+^ T cells specifically recognize and lyse MTB-infected cells in an HLA-I-restricted manner, so a key step in this research is to identify the epitopes of these antigens.

The majority of anti-TB drugs are pumped out by the efflux pumps of MTB, and some efflux pump proteins are highly expressed or mutated in drug-resistant MTB, including MDR-TB, XDR-TB, and TDR-TB. To combat drug-resistant MTB, several epitopes of the proteins involved in TB drug resistance have been identified. In our previous study, we identified several epitopes of MTB proteins, such as the secreted protein CFP21, an RD-11 region-encoded protein Rv3425, overexpression efflux pump proteins Rv1410c, Rv1258c, Rv2686c-Rv2687c-Rv2688c, and Rv2937 (DrrB), and the mutant proteins KatG and InhA [32, 37, 38]. Previous studies have also reported that the overexpression of different efflux pump genes is associated with the resistance of clinical isolates of MTB to many drugs [25]. The overexpression of efflux pump Rv1218c is responsible for the efflux of a wide variety of drugs, including isoniazid (INH) and rifampicin (RIF) [21, 39]. Studies have shown that Rv2477c has strong sensitive ATPase activity to orthovanadate, so Rv2477c may be involved in mycobacterial protein translation and in its resistance to tetracyclines and macrolides [40]. In the present study, we identified the HLA-A2-restricted epitopes on 10 selected antigens (Rv0194, Rv0933 (PstB), Rv1218c, Rv1819c, Rv2209, Rv2477c, Rv1877, Rv2459c, Rv2994, and Rv3728, members of the ABC and the MFS families). 29 epitopes were selected and synthesized. After the exclusion of several epitopes based on hydrophobicity, steric hindrance, and temperature, 17 epitopes were selected for further analysis (Tables 1 and 2). The T2A2 binding affinity of the epitopes was measured, and FI > 1.5 was determined for 14 of the 17 peptides. The stability of the 14 peptide/MHC complexes was then tested, and the DC_50_ values for 6 of 14 peptides exceeded 4 h (Table 2 and Figure 1). Based on the results for T2A2 binding affinity and stability, the immunogenic activity of these six peptides (Rv1218c-p24, Rv1218c-p200, Rv1218c-p158_,_ Rv2209-p158, Rv2477c-p182, and Rv2459c-p469) was tested *in vitro* and *in vivo*.

The PPD test (tuberculin skin test) is broadly used to diagnose whether the human body is infected with MTB and the degree of the immunological response to infection, but the sensitivity and specificity of the PPD test can be reduced by previous BCG vaccination, severe malnutrition, malignant tumors, immunodeficiency diseases, and so on. Lalvani et al. first applied the enzyme-linked immunospot experimental technique to the diagnosis of TB [41]. In the present study, we examined whether these peptides can distinguish PPD^+^ from PPD^−^ donors. The results of an *in vitro* ELISPOT assay (Figures 3(a) and 3(b)) showed that Rv2459-p469-, Rv1218c-p24-, and Rv2477c-p182-induced CTLs from PPD^+^ donors contained more CD8^+^ IFN-*γ*^+^ T cells than those from PPD^−^ donors, indicating that the IFN-*γ* production triggered by these three peptides (Rv2459c-p469, Rv1218c-p24, and Rv2477c-p182) distinguished the PPD^+^ donors from the PPD^−^ donors. This phenomenon may have utility as a more accurate and convenient index of MTB infection than the PPD test. Analysis of the *in vitro* LDH lysis activity of these three peptides (Figure 3(c)) showed that Rv1218c-p24 and the Rv2477c-p182-induced T cells lysed peptide-loaded T2A2 cells, so these two peptides were selected for an *in vivo* assay. Several studies have shown that complete Freund's adjuvant (CFA) significantly enhances the phagocytosis by macrophage in mice, and that this is closely associated with the improved immune response conferred by adjuvants [42, 43]. In an *in vivo* experiment in mice, the CFA adjuvant was subcutaneously injected first into the mouse, then the antigenic peptide was subcutaneously emulsified with IFA. The immunogenic effect of the antigenic peptide was greater in the mice injected with CFA than in the mice injected with only IFA. This suggests that CFA effectively enhanced the immune activity of the antigenic peptides in the study of the epitope peptide immunization of MTB. In addition, the IFA and CFA were used to immunize the HLA-A2.1/K^b^ transgenic mouse, and for further human beings use, other adjuvants should be used for TB vaccine.

In conclusion, using a binding affinity experiment, stability analysis, and the assessment of immune activity *in vivo* and *in vitro*, we identified two advantages of epitopes Rv1218c-p24 and Rv2477c-p182 that effectively stimulate the immune response to MTB. These two peptides may be useful as accurate and convenient indices of MTB infection and may lay a foundation for developing multivalent peptide vaccines against drug-resistant MTB.

## Figures and Tables

**Table 1:** Prediction scores of HLA-A2-restricted peptides derived from efflux pump antigens.

Antigen	Position	Sequence	Scores		
			SYFPEITHI	BIMAS	NetCTL-1.2
Rv0194	p160	LLAVLLVPV	31	271.948	1.1865
Rv0933	p186	LLDEPTSAL	28	59.558	1.1356
	p208	SLADRLTVI	27	40.336	1.28
Rv1218c	p24	ALDGLDLTV	28	27.821	1.2377
	p200	ALCEKVTII	27	40.336	0.9255
	p158	LLDEPSSGL	26	59.558	0.988
	p280	SLVSQPPTL	25	21.362	1.0547
	p165	GLDPLMENV	24	31.354	1.1238
Rv1819c	p74	LLLSVVLAV	30	1006.209	1.3652
	p70	MLGVLLLSV	28	271.948	1.2016
	p122	WMSIGVFSV	26	3082.436	1.2808
Rv2209	p158	VLATGVTLV	29	271.948	1.2373
Rv2477c	p182	LLDEPTNHL	28	59.558	0.9542
	p316	RLGNVVVEV	28	159.97	1.1527
	p362	TLFKTIVGL	28	181.794	1.1419
	p265	RLTEELAWV	27	3607.314	1.2421
	p173	LLLSKPDLL	25	65.841	0.989
Rv1877	p71	YLLGGTVVV	32	485.348	1.4044
	p95	LLGSVVVFV	28	1495.716	1.166
	p215	YLGILVIAV	28	735.86	1.217
	p295	AMLGALTFV	28	2351.109	1.3441
Rv2994	p223	LMMPQTVTV	29	315.959	1.407
	p100	SLLWIGVFL	26	434.725	1.2626
	p307	VLLMIAISV	25	437.482	1.2895
Rv3728	p264	LLAAAVAMV	30	271.948	1.2172
	p313	YLLTHVLFL	29	4599.389	1.5011
	p110	ILGVALFTV	28	1577.3	1.2034
	p54	FLDSTIVNV	27	294.344	1.5013
Rv2459c	p469	VLAADAVFV	25	650.311	1.1335

**Table 2:** The data of ESI-MS and the binding affinity and stability of peptide/HLA-A^∗^0201 complex.

Antigen	Peptide	ESI − MS[M ± H]^±^		MFI	FI^b^	DC_50_^c^
		Calculated	Observed			
Cell	-	-	-	5.64	-	
Background	-	-	-	131	-	-
Rv0194	p160	936.25	—^a^	ND	ND	ND
Rv0933	p186	958.08	959.90	465	2.52	<2 h
	p208	987.16	988.57	479	2.59	<2 h
Rv1218c	p24	916.04	916.89	569	3.32	>4 h
	P200	989.24	989.78	366	1.75	>4 h
	p158	930.02	930.85	439	2.35	>4 h
	p280	941.09	939.98	353	1.62	<2 h
	p165	987.14	987.69	479	2.6	> 2 h
Rv1819c	p74	926.21	—^a^	ND	ND	ND
	p70	944.25	—^a^	ND	ND	ND
	p122	1025.24	1026.6	177	0.35	ND
Rv2209	p158	872.07	873.4	462	2.54	>4 h
Rv2477c	p182	1051.16	1051.77	379	1.87	>6 h
	p316	984.16	984.8	348	1.58	>2 h
	p362	991.24	991.85	367	1.72	> 2 h
	p265	1116.28	1116.87	315	1.36	ND
	p173	1011.27	1011.89	272	1.15	ND
Rv1877	p71	920.12	919.59	419	2.17	>2 h
	p95	932.17	—^a^	ND	ND	ND
	p215	960.22	—^a^	ND	ND	ND
	p295	922.16	—^a^	ND	ND	ND
Rv2994	p223	1019.29	1020.62	430	2.28	<2 h
	p100	1047.3	—^a^	ND	ND	ND
	p307	958.27	—^a^	ND	ND	ND
Rv3728	p264	858.11	—^a^	ND	ND	ND
	p313	1118.38	—^a^	ND	ND	ND
	p110	932.17	—^a^	ND	ND	ND
	p54	1007.15	—^a^	ND	ND	ND
Rv2459c	p469	904.07	905.42	440	2.36	>4 h
COX-2^d^	p321	999.6	1000.3	408	2.11	>4 h

^a^Purification failed. ^b^FI = [mean fluorescence intensity (MFI) of the peptide − MFI of the background]/[MFI of the background]. ^c^DC_50_: estimate of the time required for the loss of 50% of the peptide/HLA-A^∗^0201 complexes stabilized at time 0 h. ^d^Positive control peptide. ND: not determined. Background group was added with the corresponding solution buffer.

## Data Availability

Correspondence and requests for materials should be addressed to Prof. Yahong Wu (yahongwu@zzu.edu.cn).
